# The Complete Mitochondrial Genome of *Ophioglossum vulgatum* L. Is with Highly Repetitive Sequences: Intergenomic Fragment Transfer and Phylogenetic Analysis

**DOI:** 10.3390/genes13071287

**Published:** 2022-07-21

**Authors:** Jing Hao, Yingyi Liang, Yingjuan Su, Ting Wang

**Affiliations:** 1 College of Life Sciences, South China Agricultural University, Guangzhou 510642, China; jinghaoscau@163.com (J.H.); yyliangchn@163.com (Y.L.); 2 School of Life Sciences, Sun Yat-sen University, Guangzhou 510275, China; 3 Research Institute of Sun Yat-sen University in Shenzhen, Shenzhen 518057, China

**Keywords:** *Ophioglossum vulgatum*, mitochondrial genome, phylogeny, fragment transfers, repetitive sequences

## Abstract

Many plant mitochondrial (mt) genomes have been sequenced but few in ferns. *Ophioglossum vulgatum* represents a typical species of fern genus *Ophioglossum* with medicinal and scientific value. However, its mt genome structure remains to be characterized. This study assembled and annotated the complete *O. vulgatum* mt genome and presented its structural characters and repeat sequences firstly. Its mt and chloroplast (cp) transfer sequences were explored, and the phylogenetic significance of both mt and cp genomes was also evaluated at the family level. Our results showed that the complete mt genome of *O. vulgatum* is a single circular genome of 369,673 bp in length, containing 5000 dispersed repetitive sequences. Phylogenetic trees reconstructed from cp and mt genomes displayed similar topologies, but also showed subtle differences at certain nodes. There exist 4818 bp common gene fragments between cp and mt genomes, of which more than 70% are located in tRNA intergenic regions (in mt). In conclusion, we assembled the complete mt genome of *O. vulgatum*, identified its remarkable structural characters, and provided new insights on ferns. The complementary results derived from mt and cp phylogeny highlighted that some higher taxonomic-level phylogenetic relationships among ferns remain to be resolved.

## 1. Introduction

The mitochondrion is a semi-autonomous organelle with a genetic system independent of the cell nucleus [1,2]. The mitochondrion plays an important role in metabolic processes. It provides energy for eukaryotes and is the site of ATP synthesis [3]. The mitochondrial (mt) genomes of plants are large and complex, with a length of 200–2000 kbp and a variable structure [4]. The mt genome structure is often described as circles, but its real structure appear diversely as circular, linear, and complex branched [5] (e.g., *Cucumis sativus* has three circular chromosomes that replicate completely or largely autonomously [6]; and *Lactuca sativa* has a variety of linear, circular and branched mt genome structures [7]). The complex and variable mt genome structures in plants may be caused by large numbers of repetitive sequences [8]. The sequences enable the mediation of inter- and intramolecular homologous recombination within the mt genome, leading to structure diversity [9]. It is known that the level of repetitive sequence-mediated recombination is also varied [10], with rates associated with repeat sizes [8].

In addition, nuclear and chloroplast (cp) DNA transfers into mt DNA in plants are more frequent in comparison with other eukaryotes [11,12,13,14]. Generally, transfers of mt sequences into cp genome occur much less frequently [15,16]. Typical events of intergenomic fragment transfers detected in higher plants include transfers of mt *coxII* gene into nucleus via RNA mediation [11] and cp photosynthesis-related genes or fragments into nuclear or mt genomes (Orobanchaceae) [14,17], cp sequences into nucleus (mitochondria as a bridge, *Actinidia*) [18], and mt sequences into nucleus (*Dalbergia odorifera*) [3]. These findings suggest that intergenomic fragment transfers may reflect a common evolutionary phenomenon [14], underlining their importance for understanding plant mt genome evolution.

With the advancement of high-throughput sequencing technology, considerable organelle genome data have been applied to phylogenetic research. In particular, the whole cp and mt genome sequences have been noted to be useful barcodes [19,20]. Evolutionary rates of cp genome tend to be slightly faster than those of mt genome [21]. Additionally, cpDNA markers have been more widely used than mtDNA in plant molecular phylogenetic studies. Partially, this is due to the instability of mtDNA size, structure, and sequence content [22]. Nevertheless, the mtDNA encoded genes can be conserved [23] and have the potential to be used in addressing unsolved phylogenetic issues.

Among land plants, *Marchantia polymorpha* is the first whose mt genome was sequenced [24]. To date (as of June 2022), there are a total of 465 complete plant mt genomes that have been deposited in the National Center for Biotechnology Information (NCBI) Organelle Genome Database. Of those, however, only three are ferns. Mt genome sequence data of ferns are critical for fully understanding mt genome characters of plants, as ferns represent the sister group of seed plants. Family Ophioglossaceae is one of the most fascinating lineages among ferns. It is among the second earliest-diverging lineages of ferns and has the largest known number of nuclear chromosomes [25]. *Ophioglossum vulgatum* is a typical species of the genus *Ophioglossum* with significant medicinal and scientific value [26]. The plant is difficult to characterize at the nuclear genome level due to its large number of nuclear chromosomes (2n = 240–1140) [27]. In a previous study, we sequenced the complete cp genome of *O. vulgatum* [28], but its mt genome remains to be explored.

In this study, in order to reveal the structural features, intergenomic fragment transfers, and phylogenetic usefulness of *O. vulgatum* mt genome, we conducted the following investigations: (1) the complete mt genome sequence of *O. vulgatum* was sequenced, assembled, and annotated; (2) the structural characters and repetitive sequences of *O. vulgatum* mt genome were presented, and the phylogenetic significance of mt simple sequence repeats (SSRs) was evaluated at family level; (3) the consistency and differences in family-level phylogeny reconstructed using mt and cp gene sequences were revealed; and (4) the length, position, and potential function of the genomic fragments transferred between mt and cp genomes were characterized. 

## 2. Materials and Methods 

### 2.1. DNA Extraction, Illumina DNA Library Construction and Sequencing 

The plant materials were collected from South China Agricultural University (113°20′ E, 23°9′ N), and fresh leaves of *O. vulgatum* were selected for total DNA extraction. The total DNA was extracted using a plant DNA extraction kit according to the instructions (CWBIO CW0553, Nanjing, Jiangsu, China). After extraction, the qualified samples were used to construct a paired-end sequencing Illumina DNA library with an insert size of 350 bp. Then, the qPCR and Agilent 2100 Bioanalyzer (Agilent Technologies, Santa Clara, CA, USA) were used for quality control. Sequencing was performed on the Illumina NovaSeq6000 (Illumina, San Diego, CA, USA) high-throughput sequencing platform, and the sequencing strategy was PE150 (Pair-End 150). 

### 2.2. Assembly and Annotation of the mt Genome

High-quality clean reads were obtained after filtering raw reads generated from Illumina high-throughput sequencing. Then, we used the pair-end clean reads and our PacBio Isoform Sequencing (Iso-Seq) full-length transcriptome data to assemble and annotate the mt genome (our full-length transcriptome data was sequenced with PacBio Sequel II platform (Pacific Biosciences, Menlo Park, CA, USA); our RNA Iso-seq sequences and mRNA sequences were deposited in the NCBI Sequence Read Archive (SRA) under the study accession number PRJNA856114). The mtDNA sequence was assembled by using SPAdes v. 3.13.0 with the plasmid method and multi k-mer parameters [29]. The full-length transcripts of *O. vulgatum* and the mt genome of Polypodiopsida were used as references. Pair-end sequencing reads were then re-aligned to the assembled mt sequences to confirm the assembly and close gaps. The mRNA sequences were mapped to the mt genome with minimap2 and blastn to annotate the mt genes and identify mt RNA editing sites [30,31,32]. The tRNAscan-SE v. 2.0 software was used to predict tRNAs [33]. OGDRAW v. 1.3.1 was used to draw mt genome maps [34]. The complete mt genome was deposited in Genbank under the accession number OL800577.

### 2.3. Analysis of mt Genome Characters

We selected mt genomes of three bryophytes, three ferns, four gymnosperms, four monocotyledonous angiosperms, and four dicotyledonous angiosperms from NCBI (*O. vulgatum* mt genome was sequenced in this study) for conducting statistical analysis of mt-encoding protein genes (Table 1). DnaSP v. 6.12.03 was used to calculate nucleotide variability (Pi) values [35]. Mutation sites were mapped to the *O. vulgatum* mt genome to identify their locations.

### 2.4. Phylogenetic and Fragment Transfer Analysis of mt and cp Genomes

Mt and cp complete genomes of sampling species (Table 1) were downloaded from Genbank. PhyloSuite (v. 1.2.2, Zhang et al., Wuhan, China) were used to extract the common mt and cp CDS sequences [36]. The MAFFT (v. 7) plugin (based on codons) in PhyloSuite was used for multiple sequence alignment [37]. Then, concatenated data sets of cp and mt common genes were constructed separately. The Gblocks (v. 0.91b) plugin in PhyloSuite was used to optimize protein sequence alignments [38]. A maximum likelihood (ML) phylogenetic tree was constructed using the IQtree (v. 1.6.8) plugin in PhyloSuite (Ultrafast bootstrap, bootstrap = 1000, three bryophytes set as outgroups) [39,40]. For mtDNA, we chose GTR + F + G4 (“GTR” means general time reversible model with unequal rates and unequal base freq, “+F” means empirical base frequencies, and “+G4” means discrete γ model with the number of categories as G4) as the best-fit model based on Bayesian information criterion (BIC). For cpDNA, we selected GTR + F + R3 (“+ R3” means FreeRate model that generalizes the + G model by relaxing the assumption of γ-distributed rates with the number of categories R3) as the best-fit model. In addition, the MrBayes v. 3.2.6 plugin in PhyloSuite was used to conduct the Bayesian inference (BI) [41]. The web tool ITOL v. 5 was used to beautify phylogenetic trees [42]. Homologous sequences between cp and mt genomes were searched by Blast in TBtools (e-value = 1 × 10^−5^, Num of Hits = 50,000, and Num of Aligns= 25,000) [43]. The Circos plot was drawn using TBtools.

### 2.5. Identification and Analysis of Repetitive Sequences

SSRs were identified using MISA-web (version 2.1) [44]. Ten, six, five, five, five, and five repeat units were set as the minimum thresholds for the identification of mono-, di-, tri-, tetra-, penta-, and hexa-motif microsatellites, respectively. The maximum length between two SSRs in the composite SSR was set as 0 bp. The web tool REPuter program was used to search the dispersed repeats (Hamming distance = 3; maximum computed repeats = 5000; minimal repeat size = 30; forward (F), reverse (R), complement (C), and palindromic (P) repeats were all permitted) [45,46]. An advanced Circos plot was drawn with TBtools. Finally, we evaluated the phylogenetic significance of SSRs by examining mtSSR characteristics in the context of mt ML tree.

## 3. Results

### 3.1. Characters of the Complete mt Genome of O. vulgatum

**Figure 1:**
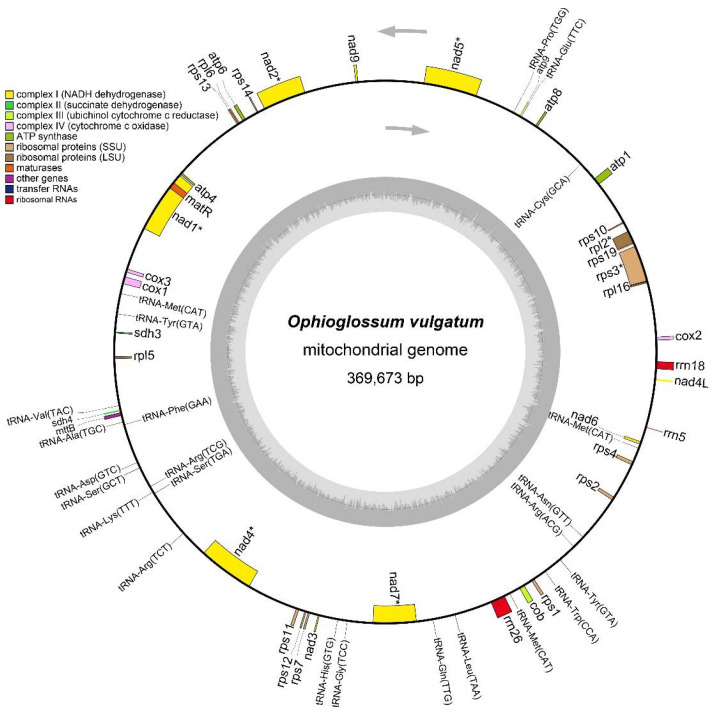
Mitochondrial genome map of *O. vulgatum*. The total length of the mitochondrial genome is 369,673 bp. Genes shown on the inside of the circle are transcribed clockwise, whereas those on the outside are transcribed counter-clockwise. Genes containing introns are marked by an asterisk (*).

We successfully assembled the complete mt genome of *O. vulgatum*. It is a single circular genome with a length of 369,673 bp and a total GC content of 52.14% (Figure 1). We annotated 64 genes including 37 protein coding genes, 24 tRNA genes, and three rRNA genes (Table 2). The longest gene is *rrn26* (3283 bp), located in the positive strand; and the shortest are *tRNA-Gly* (71 bp) and *tRNA-Cys* (71 bp), located in the positive and negative strands, respectively. Among the 37 protein coding genes, two (*rpl2*, *rps3*) contain one intron, two (*nad4*, *nad7*) have three introns, and three (*nad1*, *nad2*, *nad5*) contain four introns. RNA editing occurred in 26 genes, accounting for 70.27% of all protein-coding genes. Besides *O. vulgatum*, we selected three bryophytes, two ferns, four gymnosperms, and four monocotyledonous and four dicotyledonous angiosperms to perform analysis of mt protein coding genes (Table 2 and Table S1). Four *ccm* (*c*ytochrome *c m*aturation) genes (*ccmB*, *ccmC*, *ccmFC*, and *ccmFN*) were lost from *O. vulgatum* in comparison with most selected plants. The ribosomal protein 6 gene (*rpl6*) was present in three ferns (including *O. vulgatum*) compared with other selected seed plants. The results were consistent with the analysis of its related species *O. californicum* [10]. We speculated that the entire cytochrome *c* pathway may be lost in *O. vulgatum*.

### 3.2. Comparative mt Genomic Analysis between O. vulgatum and O. californicum

**Figure 2:**
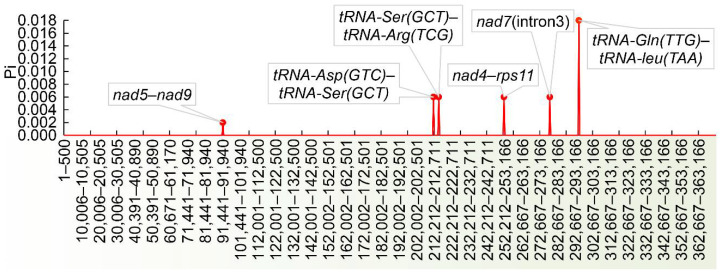
Hypervariable regions between *O. vulgatum* and *O. californicum*. The horizontal axis shows the location information for *O. vulgatum* mitochondrial genome, and the vertical axis shows the Pi values.

We compared the mt genomes of *O. californicum* and *O. vulgatum* (Figure 2). There were six hypervariable regions between the two species. The maximum Pi value was 0.018, which was derived from the intergenic region between *tRNA-Gln (TTG)* and *tRNA-Leu (TAA)* in *O. vulgatum*. The minimum Pi value was 0.002, which was from the intergenic region between *nad5* and *nad9*. The remaining four variable loci were located as follows: the intergenic region between *tRNA- Asp (GTC)* and *tRNA-Ser (GCT)*, the intergenic region between *tRNA-Ser (GCT)* and *tRNA-Arg (TCG)*, the intergenic region between *nad4* and *rps11*, and the intron 3 of *nad7*. Our results revealed that there were six hypervariable regions between the two species, which were mainly located in mt intergenic regions.

### 3.3. Comparative Phylogenetic Analysis Based on mt and cp Genomic Sequences

**Figure 3:**
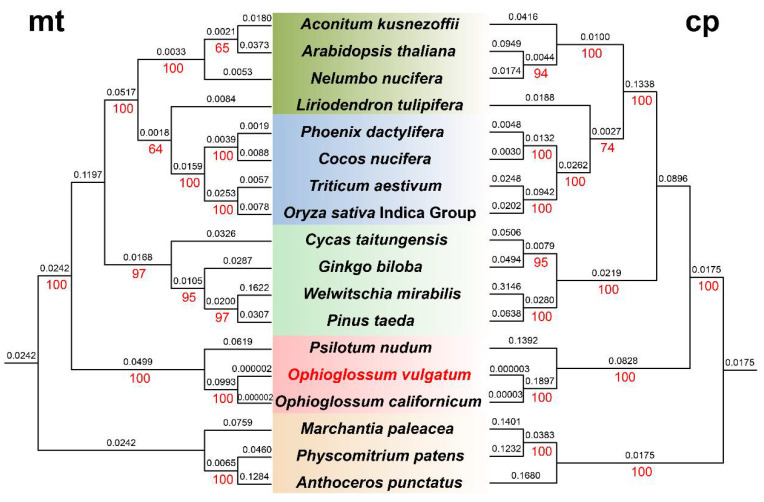
Maximum likelihood (ML) trees reconstructed by using concatenated datasets of common mitochondrial (left) and chloroplast (right) genes of representative species. Red numbers below the branches are bootstrap values. Values above the branches are branch lengths. The boxes with different colors represent different plant groups (the bryophytes, ferns, gymnosperms, monocots, and dicots).

We concatenated all common coding genes from mt and cp genomes and used the matrices to infer phylogenetic trees based on mt and cp data, respectively. ML trees are shown in Figure 3, and BI trees are shown in Figure S1 (as ML and BI trees have similar topologies, we focused on the ML tree here). Phylogenetic trees reconstructed from cp and mt data have roughly the same topology for the major branches. Bryophytes, ferns, gymnosperms, and angiosperms were clustered in four branches in both cp and mt trees. Branch lengths in the cp tree tended to be longer than those in the mt tree (except for *C. nucifera*). Two *Ophioglossum* species formed a monophyletic clade in both mt and cp trees, and *P. nudum* diverged earlier. In the mt tree, bryophytes formed a topology as ((*P. patens*, *A. punctatus*), *M. polymorpha*). By contrast, in the cp tree, *A. punctatus* was the sister to a clade containing *P. patens* and *M. polymorpha*. For gymnosperms, the cp tree showed that *C. taitungensis* and *G. biloba* clustered together. However, the mt tree showed that the two species did not. Both mt and cp trees showed that *L. tulipifera* and the other four monocots were clustered together, but with weak support (bootstrap = 64/74). The remaining dicots formed a clade. Their branching pattern in the cp tree was ((*N. nucifera*, *A. thaliana*), *A. kusnezoffii*); but in the mt tree, it was (*N. nucifera*, (*A. thaliana*, *A. kusnezoffii*)), which was consistent with the APG IV system [47]. The phylogenetic results indicated that the phylogenetic trees reconstructed from cp and mt genome sequences had roughly the same topologies, but there existed subtle differences in the branching structure. The complementary information provided by mt and cp trees highlighted that some significant phylogenetic relationships remain to be clarified.

### 3.4. Intergenomic Fragment Transfers between mt and cp Genomes

**Figure 4:**
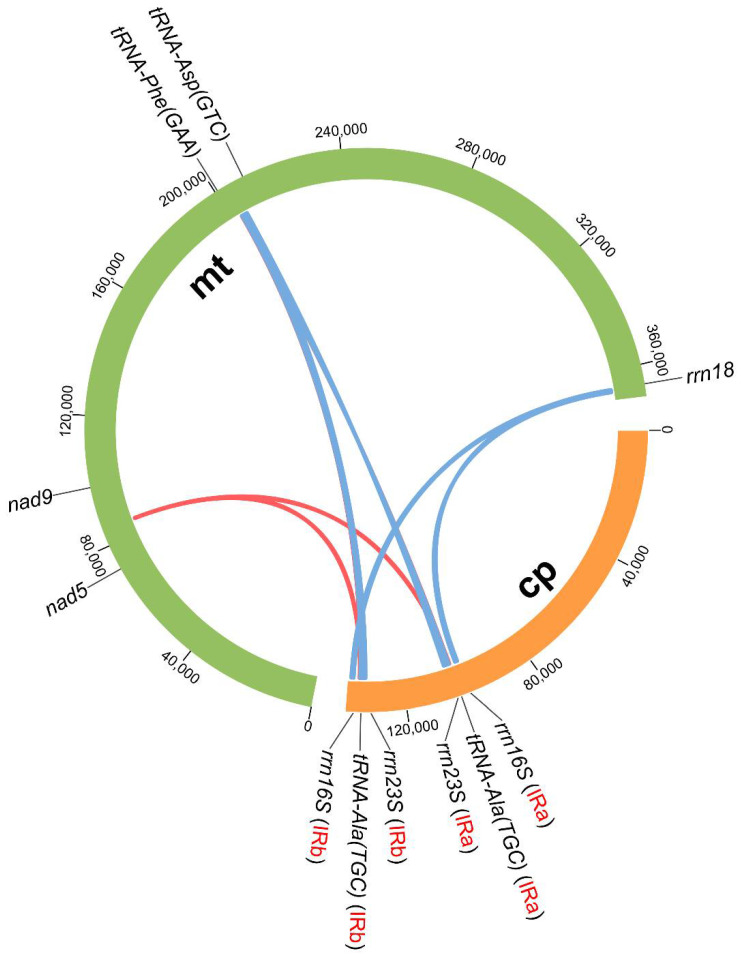
Locations of the transferred fragments between mitochondrial and chloroplast genomes. Green circle represents mitochondrial genome, and orange circle chloroplast genome. Blue and red lines inside the circle correspond to fragment lengths more or less than 100 bp, respectively. Ends of the same line indicate the location of common gene fragments.

We identified 14 common gene fragments between the cp and mt genomes of *O. vulgatum* (Figure 4 and Table S2). The fragment lengths ranged from 52 to 683 bp, with a total length of 4818 bp. In the cp genome, the common gene fragments were symmetrically distributed in IRa (Inverted repeat a) and IRb regions, located in *rrn16S*, *rrn23S*, *tRNA-Ala (TGC)*, and the *tRNA-Ala (TGC)*–*rrn23S* intergenic region. In the mt genome, the common gene fragments were located in *rrn18*, *nad5*–*nad9* intergenic region, and *tRNA-Asp (GTC)*–*tRNA-Phe (GAA)* intergenic region. We speculated that there existed fragment transfers between mitochondrion and chloroplast genomes of *O. vulgatum*.

### 3.5. Analysis of Repetitive Sequences

**Figure 5:**
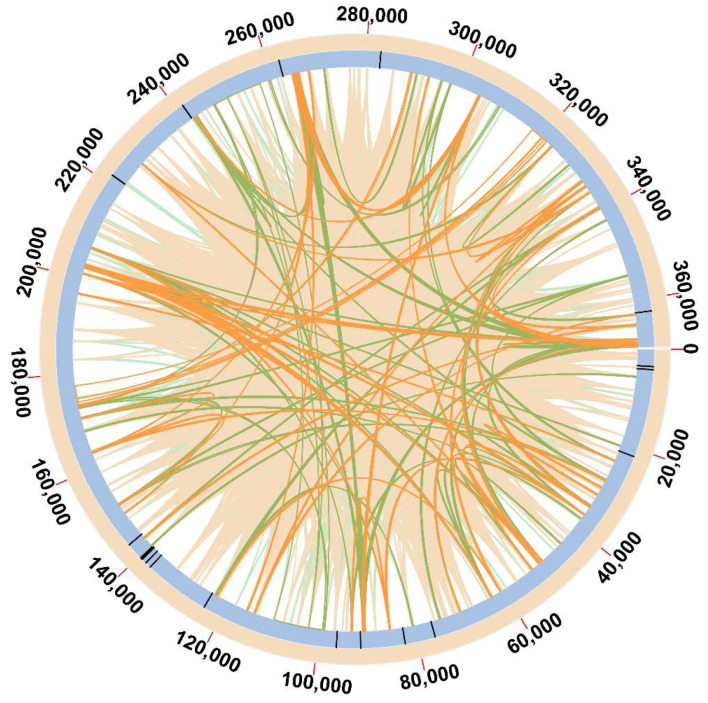
Simple sequence repeats (SSRs) and dispersed repetitive sequences in the mitochondrial genome of *O. vulgatum*. Black lines on the blue circle indicate the SSR locations. Lines inside the circle show the distribution of dispersed repetitive sequences; green lines represent forward (F) repeats, and orange lines represent palindromic (P) repeats (light green and orange lines correspond to lengths less than 200 bp).

A total of 5000 dispersed repetitive sequences were detected in the *O. vulgatum* mt genome. Their lengths ranged from 108 to 7435 bp. The sequences contained 2408 palindromic (P) repeats and 2592 forward (F) repeats. There were seven repeats larger than 1000 bp. Positions of the repeats are shown in Figure 5 and Table S3. In addition, a total of 20 SSRs were detected in the *O. vulgatum* mt genome, with sizes ranging from 10 to 48 bp (Figure 5 and Table S4). The SSR types were as follows: seven mononucleotides (mono-), with G/C as repeating unit; seven dinucleotides (di-), with TC/CT (5), AC (1), and GA (1) as repeating units; two trinucleotides (tri-), with GTG/TTA as repeating unit; one tetranucleotide (tetra-) with AAAG as repeating unit; one pentanucleotide (penta-), with AAGTA as repeating unit; and two hexanucleotides (hexa-), with TCCAAC as repeating unit. Most SSRs were located in intergenic regions, except for four SSRs that were in the introns of *nad4*, *nad5*, and *nad7*.

**Figure 6:**
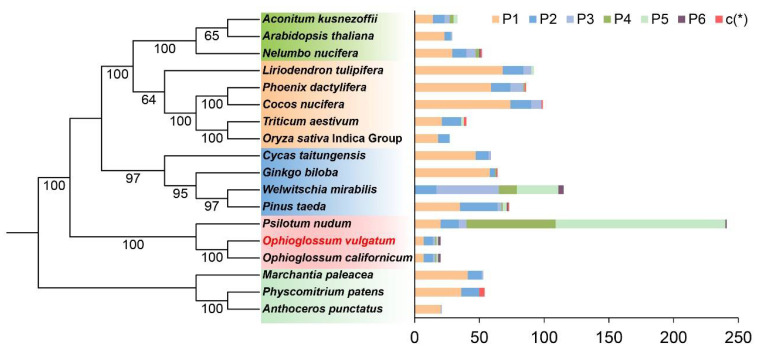
Simple sequence repeats (SSRs) characteristic of 18 representative species and their association with phylogeny. P1, p2, p3, p4, p5, p6, and c (*) represent mono-, di-, tri-, tetra-, penta-, hexa-, and compound SSRs, respectively. Clades in the tree are highlighted with color boxes. Horizontal axis in the right figure presents the number of different SSR types.

Previously, we noted that distribution characteristics of cp SSRs may provide useful phylogenic information at the genus level [48]. Here, we further evaluated the phylogenetic significance of mt SSRs based on the mt tree (Figure 6). No association was found between the distribution characteristics of mt SSRs and phylogenetic relationships at family level. Nevertheless, *O. vulgatum* and *O. californium* showed consistent mtSSR distribution patterns. *W. mirabilis* has no mono- repeating units. *L. tulipifera* is a dicot, and its mtSSR distribution showed similarity with two Arecaceae monocots. Noteworthily, *L. tulipifera* and the two Arecaceae monocots were clustered in the same clade in the mt tree.

## 4. Discussion

It has been well noted that a considerable number of mt genes have been lost or functionally transferred during mt evolution [11,49]. In this study, *O. vulgatum* was found to be lacking four mt *ccm* genes (*ccmB*, *ccmC*, *ccmFC*, and *ccmFN*) in comparison with most other sampling plants. To consolidate the mt gene loss, we also mapped the four *ccm* gene sequences to our full-length transcriptome datasets. No corresponding transcripts were detected. Proteins encoded by the mt *ccm* genes function in the maturation pathway of cytochrome *c* and are important electron transporters in the mt respiratory chain of plants [50]. Moreover, the *ccm* genes were also detected as lost in the bryophyte *A. punctatus* but not in *P. patens* and other examined plants. We speculated the functions of the mt *ccm* genes might have been replaced by nuclear counterparts [49]. On the other hand, a *rpl6* gene addition was observed in the mt genomes of three ferns (*P. nudum*, *O. vulgatum*, and *O. californicum*). It cannot be excluded that the *rpl6* gene may have been present in the ancestor of vascular plants and functionally transferred to the nucleus or lost in certain lineages during evolution [10,11,49].

There exist frequent gene transfers from cp to mt genomes in plants [15]. For example, photosynthesis-related genes have been observed to be transferred from cp to mt genomes in Orobanchaceae [14,17]. Here, we identified 14 common gene fragments between the cp and mt genomes in *O. vulgatum*, with a total length of 4818 bp (as shown in mt genome, Table S2). The results underscore extensive gene or fragment transfers between the two *O. vulgatum* genomes, but we were unable to determine the transfer direction. Previous studies indicate that the mt genome tends to takes up cp sequences rather than the opposite [15,16]. Thus, we hypothesize that the common fragments detected in *O. vulgatum* could be generated by cp to mt transfers. Importantly, Miyata et al. (1998) detected some plastid-derived sequences in the rice mt genome encoding tRNA genes [51]. Here, we also noticed that more than 70% of the transferred fragments were located in tRNA intergenic regions in the *O. vulgatum* mt genome. In addition, Notsu et al. (2002) showed that cp fragments may be first integrated into mt genome and then transferred to the nucleus (i.e., the mt genome functions as an intermediate) [52].

This study revealed considerable repetitive sequences in the mt genome of *O. vulgatum* like in other plants [53]. A total of 5000 dispersed repetitive sequences were detected with lengths ranging from 108 to 7435 bp. Repetitive sequences may mediate frequent recombination, facilitating genome diversity [9]. However, Guo et al. (2017) noted that the mt genomes of ferns *O. californicum* and *P. nudum* maintain an extremely low level of active recombination, although their mt genomes are highly repetitive [10]. This highlights the possibility that the frequency of repetitive sequence-mediated mt recombination may vary greatly across plant lineages. Additionally, we also identified a total of 20 mtSSRs in *O. vulgatum* with sizes ranging from 10 to 48 bp. Previously, the distribution characteristics of cp SSRs were found to be capable of providing phylogenetic signals at the genus level [48]. In this study, similar characteristics were indeed observed in the mtSSRs of *O. vulgatum* and *O. californium*. However, no association was found between the mtSSR characteristics and phylogenetic relationships at the family level. It is noteworthy that mtSSRs of the dicot *L. tulipifera* show similar characteristics with monocots, and in the mt ML tree, *L. tulipifera* is found to cluster with monocots. These results indicate that the phylogenetic significance of the mtSSR patterns deserves to be further explored in the future.

The complete cp genome sequences have been extensively used to reconstruct plant phylogeny [3,21]. In contrast, mt genome sequences appear to have been relatively scarce in this respect [21]. Nevertheless, mt and cp sequences may provide complementary information for phylogenetic inference [54,55]. Here, we performed a comparative phylogenetic analysis by using the datasets constructed from the shared cp or mt genes. Similar family-level phylogenetic relationships have been reconstructed, but there are subtle differences: the phylogenetic position of *C. taitungensis* and *G. biloba* in the gymnosperms, and the position of *N. nucifera* and *L. tulipifera* in angiosperms. These inconsistencies suggest that some higher taxonomic-level phylogenetic controversies remain to be solved. They also restate the importance of facilitating plant mitochondrial phylogenomic studies.

## 5. Conclusions

Based on this study, our conclusions are as follows: (1) the complete mt genome of *O. vulgatum* is a single circular genome with extensive repetitive sequences; its mt encoded *ccm* genes are lost, and the mt genome has six intergenic regions that are hypervariable; (2) distribution characteristics of mt SSRs may not provide phylogenetic signals at the family level; (3) phylogenetic trees reconstructed from cp and mt genome sequences show roughly the same topologies, but there are subtle differences; and (4) a total of 4818 bp common gene fragments have been identified between the *O. vulgatum* cp and mt genomes. This study provides new mt genome resources on ferns and highlights the importance of reexamining plant phylogenetic relationships based on both cp and mt genome sequences.

## Figures and Tables

**Table 1:** Information on sampling species.

Names	Family	Group	Genbank Accessions (mt)	Genbank Accessions (cp)
*Marchantia paleacea*	Marchantiaceae	Bryophyte	NC_001660	NC_001319
*Physcomitrium patens*	Funariaceae	Bryophyte	NC_007945	KY126308
*Anthoceros punctatus*	Anthocerotaceae	Bryophyte	NC_049003	MN544310
*Ophioglossum californicum*	Ophioglossaceae	Fern	KX171637	NC_020147
*O. vulgatum*	Ophioglossaceae	Fern	OL800577	MZ066610
*Psilotum nudum*	Psilotaceae	Fern	KX171638, KX171639	NC_003386
*Welwitschia mirabilis*	Welwitschiaceae	Gymnosperm	NC_029130	NC_010654
*Pinus taeda*	Pinaceae	Gymnosperm	NC_039746	NC_021440
*Cycas taitungensis*	Cycadaceae	Gymnosperm	NC_010303	NC_009618
*Ginkgo biloba*	Ginkgoaceae	Gymnosperm	NC_027976	NC_016986
*Oryza sativa* Indica Group	Poaceae	Angiosperm (monocot)	NC_007886	NC_008155
*Triticum aestivum*	Poaceae	Angiosperm (monocot)	GU985444	NC_002762
*Phoenix dactylifera*	Arecaceae	Angiosperm (monocot)	NC_016740	NC_013991
*Cocos nucifera*	Arecaceae	Angiosperm (monocot)	NC_031696	NC_022417
*Liriodendron tulipifera*	Magnoliaceae	Angiosperm (dicot)	NC_021152	NC_008326
*Aconitum kusnezoffii*	Ranunculaceae	Angiosperm (dicot)	NC_053920	KT820671
*Nelumbo nucifera*	Nelumbonaceae	Angiosperm (dicot)	NC_030753	NC_025339
*Arabidopsis thaliana*	Brassicaceae	Angiosperm (dicot)	Y08501	NC_000932

**Table 2:** Mitochondrial protein-coding genes of *O. vulgatum*.

Gene Type	Gene Name
ATPase subunits	*atp1*, *atp4*, *atp6*, *atp8*, *atp9*
Apocytochrome *b*	*cob*
Cytochrome *c* oxidase subunits	*cox1*, *cox2*, *cox3*
Ribosomal proteins	*rpl2*, *rpl5*, *rpl6*, *rpl16*, *rps1*, *rps2*, *rps3*, *rps4*, *rps7*, *rps10*, *rps11*, *rps12*, *rps13*, *rps14*, *rps19*
Maturase	*matR*
Sec-independent protein translocase protein	*mttB*
NADH dehydrogenase subunits	*nad1*, *nad2*, *nad3*, *nad4*, *nad4L*, *nad5*, *nad6*, *nad7*, *nad9*
Succinate dehydrogenase cytochrome subunits	*sdh3*, *sdh4*

## Data Availability

The complete mt genome was deposited in Genbank of NCBI under the accession number OL800577. The data supporting the findings of this study are available from the corresponding author upon reasonable request.

## Supplementary Materials

The following supporting information can be downloaded at: https://www.mdpi.com/article/10.3390/genes13071287/s1, Figure S1: The phylogenic Bayesian inference trees of mt (left) and cp (right) based on common gene datasets of selected species. The numbers beside the branches represent the posterior probability. The different color boxes represent different plant groups (Bryophytes, ferns, gymnosperms, monocots, and dicots); Table S1: Statistical information on mitochondrial genes of 18 species; Table S2: Location information on the common fragments between mitochondrial and chloroplast genomes; Table S3: Locations of the dispersed repetitive sequences in the mitochondrial genome of *O. vulgatum*; Table S4: SSR sequences and location information (IGS, intergenic spacer; p1, p2, p3, p4, p5, and p6 represent mono-, di-, tri-, tetra-, penta-, and hexa-SSRs, respectively).

## References

[1] Handa H. (2003). The complete nucleotide sequence and RNA editing content of the mitochondrial genome of rapeseed (*Brassica napus* L.): Comparative analysis of the mitochondrial genomes of rapeseed and *Arabidopsis thaliana*. Nucleic Acids Res..

[2] Zhang X., Zhang R., Hou S., Shi J., Guo S. (2011). Research progress on mitochondrial genome of higher plant. J. Agric. Sci. Technol..

[3] Hong Z., Liao X., Ye Y., Zhang N., Yang Z., Zhu W., Gao W., Sharbrough J., Tembrock L.R., Xu D. (2021). A complete mitochondrial genome for fragrant Chinese rosewood (*Dalbergia odorifera*, Fabaceae) with comparative analyses of genome structure and intergenomic sequence transfers. BMC Genom..

[4] Morley S.A., Nielsen B.L. (2017). Plant mitochondrial DNA. Front. Biosci..

[5] Jackman S.D., Coombe L., Warren R.L., Kirk H., Trinh E., MacLeod T., Pleasance S., Pandoh P., Zhao Y., Coope R.J. (2020). Complete mitochondrial genome of a gymnosperm, sitka spruce (*Picea sitchensis*), indicates a complex physical structure. Genome Biol. Evol..

[6] Alverson A.J., Rice D.W., Dickinson S., Barry K., Palmer J.D. (2011). Origins and recombination of the bacterial-sized multichromosomal mitochondrial genome of cucumber. Plant Cell.

[7] Kozik A., Rowan B.A., Lavelle D., Berke L., Schranz M.E., Michelmore R.W., Christensen A.C. (2019). The alternative reality of plant mitochondrial DNA: One ring does not rule them all. PLoS Genet..

[8] Maréchal A., Brisson N. (2010). Recombination and the maintenance of plant organelle genome stability. New Phytol..

[9] Lonsdale D.M., Brears T., Hodge T.P., Melville S.E., Rottmann W.H. (1988). The plant mitochondrial genome: Homologous recombination as a mechanism for generating heterogeneity. Philos. Trans. R. Soc. Lond. B.

[10] Guo W., Zhu A., Fan W., Mower J.P. (2017). Complete mitochondrial genomes from the ferns *Ophioglossum californicum* and *Psilotum nudum* are highly repetitive with the largest organellar introns. New Phytol..

[11] Nugent J.M., Palmer J.D. (1991). RNA-mediated transfer of the gene *coxII* from the mitochondrion to the nucleus during flowering plant evolution. Cell.

[12] Kubo T., Newton K.J. (2008). Angiosperm mitochondrial genomes and mutations. Mitochondrion.

[13] Lei B., Li S., Liu G., Wang Y., Su A., Hua J. (2012). Evolutionary analysis of mitochondrial genomes in higher plants. Mol. Plant Breed..

[14] Choi K.S., Park S. (2021). Complete plastid and mitochondrial genomes of *Aeginetia indica* reveal intracellular gene transfer (IGT), horizontal gene transfer (HGT), and cytoplasmic male sterility (CMS). Int. J. Mol. Sci..

[15] Hao W., Palmer J.D. (2009). Fine-scale mergers of chloroplast and mitochondrial genes create functional, transcompartmentally chimeric mitochondrial genes. Proc. Natl. Acad. Sci. USA.

[16] Smith D.R. (2011). Extending the limited transfer window hypothesis to inter-organelle DNA migration. Genome Biol. Evol..

[17] Cusimano N., Wicke S. (2016). Massive intracellular gene transfer during plastid genome reduction in nongreen Orobanchaceae. New Phytol..

[18] Wang S., Li D., Yao X., Song Q., Wang Z., Zhang Q., Zhong C., Liu Y., Huang H. (2019). Evolution and diversification of kiwifruit mitogenomes through extensive whole-genome rearrangement and mosaic loss of intergenic sequences in a highly variable region. Genome Biol. Evol..

[19] Nock C.J., Waters D.L., Edwards M.A., Bowen S.G., Rice N., Cordeiro G.M., Henry R.J. (2011). Chloroplast genome sequences from total DNA for plant identification. Plant Biotechnol. J..

[20] Asaf S., Khan A.L., Khan A.R., Waqas M., Kang S.M., Khan M.A., Shahzad R., Seo C.W., Shin J.H., Lee I.J. (2016). Mitochondrial genome analysis of wild rice (*Oryza minuta*) and its comparison with other related species. PLoS ONE.

[21] Small R.L., Cronn R.C., Wendel J.F. (2004). Use of nuclear genes for phylogeny reconstruction in plants. Aust. Syst. Bot..

[22] Duminil J., Besnard G. (2021). Utility of the mitochondrial genome in plant taxonomic studies. Methods Mol. Biol..

[23] Tian X., Zheng J., Hu S., Yu J. (2006). The rice mitochondrial genomes and their variations. Plant Physiol..

[24] Oda K., Yamato K., Ohta E., Nakamura Y., Takemura M., Nozato N., Akashi K., Kanegae T., Ogura Y., Kohchi T. (1992). Gene organization deduced from the complete sequence of liverwort *Marchantia polymorpha* mitochondrial DNA: A primitive form of plant mitochondrial genome. J. Mol. Biol..

[25] Zhang L., Fan X.P., Petchsri S., Zhou L., Pollawatn R., Zhang X., Zhou X.M., Thi L.N., Knapp R., Chantanaorrapint S. (2020). Evolutionary relationships of the ancient fern lineage the adder’s tongues (Ophioglossaceae) with description of Sahashia gen. Nov. Cladistics.

[26] Clericuzio M., Tinello S., Burlando B., Ranzato E., Martinotti S., Cornara L., La Rocca A. (2012). Flavonoid oligoglycosides from *Ophioglossum vulgatum* L. having wound healing properties. Planta Med..

[27] Zhang X., Liu Q., Sahashi N., Wu Z., Raven P.H., Hong D. (2013). Ophioglossaceae. Flora of China.

[28] Hao J., Liang Y., Zhu M., Ping J., Feng P., Su Y., Wang T. (2021). The complete chloroplast genome of *Ophioglossum vulgatum* L. (Ophioglossaceae) and phylogenetic analysis. Mitochondrial DNA B Resour..

[29] Bankevich A., Nurk S., Antipov D., Gurevich A.A., Dvorkin M., Kulikov A.S., Lesin V.M., Nikolenko S.I., Pham S., Prjibelski A.D. (2012). SPAdes: A new genome assembly algorithm and its applications to single-cell sequencing. J. Comput. Biol..

[30] Altschul S.F., Madden T.L., Schäffer A.A., Zhang J., Zhang Z., Miller W., Lipman D.J. (1997). Gapped BLAST and PSI-BLAST: A new generation of protein database search programs. Nucleic Acids Res..

[31] Zhang Z., Schwartz S., Wagner L., Miller W. (2000). A greedy algorithm for aligning DNA sequences. J. Comput. Biol..

[32] Li H. (2018). Minimap2: Pairwise alignment for nucleotide sequences. Bioinformatics.

[33] Lowe T.M., Chan P.P. (2016). tRNAscan-SE On-line: Integrating search and context for analysis of transfer RNA genes. Nucleic Acids Res..

[34] Greiner S., Lehwark P., Bock R. (2019). OrganellarGenomeDRAW (OGDRAW) version 1.3.1: Expanded toolkit for the graphical visualization of organellar genomes. Nucleic Acids Res..

[35] Rozas J., Ferrer-Mata A., Sánchez-DelBarrio J.C., Guirao-Rico S., Librado P., Ramos-Onsins S.E., Sánchez-Gracia A. (2017). DnaSP 6: DNA sequence polymorphism analysis of large data sets. Mol. Biol. Evol..

[36] Zhang D., Gao F., Jakovlić I., Zou H., Zhang J., Li W.X., Wang G.T. (2020). PhyloSuite: An integrated and scalable desktop platform for streamlined molecular sequence data management and evolutionary phylogenetics studies. Mol. Ecol. Resour..

[37] Katoh K., Standley D.M. (2013). MAFFT multiple sequence alignment software version 7: Improvements in performance and usability. Mol. Biol. Evol..

[38] Talavera G., Castresana J. (2007). Improvement of phylogenies after removing divergent and ambiguously aligned blocks from protein sequence alignments. Syst. Biol..

[39] Minh B.Q., Nguyen M.A., von Haeseler A. (2013). Ultrafast approximation for phylogenetic bootstrap. Mol. Biol. Evol..

[40] Nguyen L.T., Schmidt H.A., von Haeseler A., Minh B.Q. (2015). IQ-TREE: A fast and effective stochastic algorithm for estimating maximum-likelihood phylogenies. Mol. Biol. Evol..

[41] Ronquist F., Teslenko M., van der Mark P., Ayres D.L., Darling A., Höhna S., Larget B., Liu L., Suchard M.A., Huelsenbeck J.P. (2012). MrBayes 3.2: Efficient Bayesian phylogenetic inference and model choice across a large model space. Syst. Biol..

[42] Letunic I., Bork P. (2021). Interactive Tree of Life (iTOL) v5: An online tool for phylogenetic tree display and annotation. Nucleic Acids Res..

[43] Chen C., Chen H., Zhang Y., Thomas H.R., Frank M.H., He Y., Xia R. (2020). TBtools: An integrative toolkit developed for interactive analyses of big biological data. Mol. Plant.

[44] Beier S., Thiel T., Munch T., Scholz U., Mascher M. (2017). MISA-web: A web server for microsatellite prediction. Bioinformatics.

[45] Kurtz S., Schleiermacher C. (1999). REPuter: Fast computation of maximal repeats in complete genomes. Bioinformatics.

[46] Kurtz S., Choudhuri J.V., Ohlebusch E., Schleiermacher C., Stoye J., Giegerich R. (2001). REPuter: The manifold applications of repeat analysis on a genomic scale. Nucleic Acids Res..

[47] Angiosperm Phylogeny Group (2016). An update of the Angiosperm Phylogeny Group classification for the orders and families of flowering plants: APG IV. Bot. J. Linn. Soc..

[48] Zhu M., Feng P., Ping J., Li J., Su Y., Wang T. (2021). Phylogenetic significance of the characteristics of simple sequence repeats at the genus level based on the complete chloroplast genome sequences of Cyatheaceae. Ecol. Evol..

[49] Adams K.L., Palmer J.D. (2003). Evolution of mitochondrial gene content: Gene loss and transfer to the nucleus. Mol. Phylogenet. Evol..

[50] Giegé P., Grienenberger J.M., Bonnard G. (2008). Cytochrome *c* biogenesis in mitochondria. Mitochondrion.

[51] Miyata S., Nakazono M., Hirai A. (1998). Transcription of plastid-derived tRNA genes in rice mitochondria. Curr. Genet..

[52] Notsu Y., Masood S., Nishikawa T., Kubo N., Akiduki G., Nakazono M., Hirai A., Kadowaki K. (2002). The complete sequence of the rice (*Oryza sativa* L.) mitochondrial genome: Frequent DNA sequence acquisition and loss during the evolution of flowering plants. Mol. Genet. Genom..

[53] Dong S., Zhao C., Chen F., Liu Y., Zhang S., Wu H., Zhang L., Liu Y. (2018). The complete mitochondrial genome of the early flowering plant *Nymphaea colorata* is highly repetitive with low recombination. BMC Genom..

[54] Van de Paer C., Bouchez O., Besnard G. (2018). Prospects on the evolutionary mitogenomics of plants: A case study on the olive family (Oleaceae). Mol. Ecol. Resour..

[55] Olson M.S., McCauley D.E. (2000). Linkage disequilibrium and phylogenetic congruence between chloroplast and mitochondrial haplotypes in *Silene vulgaris*. Proc. Biol. Sci..

