# Supplementary figures and images for: The Complete Mitochondrial Genome of Ophioglossum vulgatum L. Is with Highly Repetitive Sequences: Intergenomic Fragment Transfer and Phylogenetic Analysis
Source: Genes (Basel). 2022 Jul 21;13(7):1287. doi: 10.3390/genes13071287 (PMC9316493; doi:10.3390/genes13071287)

mt

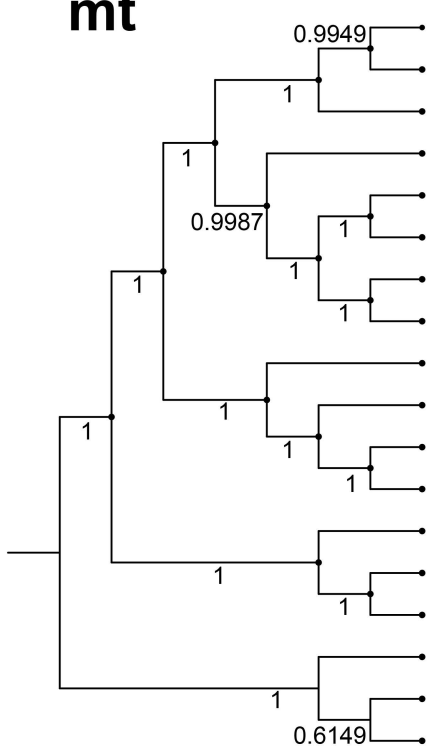

cp

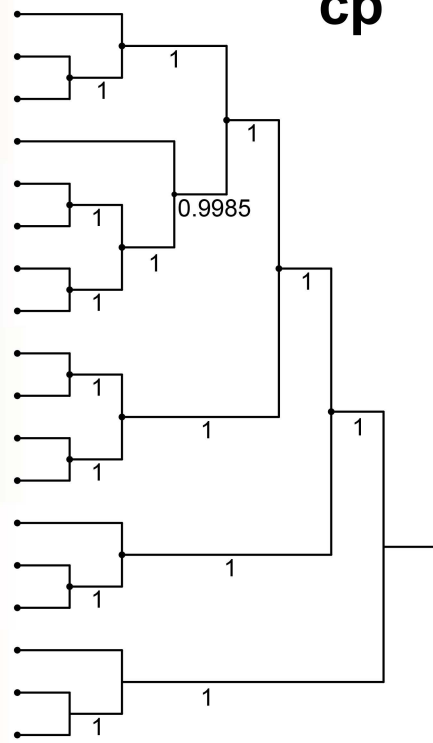

Supplement: Supplementary file 1 [file genes-13-01287-s001.zip › Figure S1.pdf]
